# Assembling Au_8_ clusters on surfaces of bifunctional nanoimmunomodulators for synergistically enhanced low dose radiotherapy of metastatic tumor

**DOI:** 10.1186/s12951-023-02279-2

**Published:** 2024-01-05

**Authors:** Rui Zhang, Mengchao Jia, Hongying Lv, Mengxuan Li, Guanwen Ding, Ge Cheng, Juan Li

**Affiliations:** 1 School of Public Health, Jilin University, Chang Chun, 130021 China; 2 Institute of Radiation Medicine, Chinese Academy of Medical Sciences & Peking Union Medical College Institute of Radiation Medicine Chinese Academy of Medical Sciences, Tianjin, 300192 China

**Keywords:** Radiosensitizer, Au clusters, Radioimmunotherapy, Metastatic tumor, Antitumor

## Abstract

**Background:**

Radiotherapy is one of the mainstays of cancer therapy and has been used for treating 65–75% of patients with solid tumors. However, radiotherapy of tumors has two limitations: high-dose X-rays damage adjacent normal tissue and tumor metastases cannot be prevented.

**Results:**

Therefore, to overcome the two limitations of radiotherapy, a multifunctional core–shell R837/BMS@Au8 nanoparticles as a novel radiosensitizer were fabricated by assembling Au_8_NCs on the surface of a bifunctional nanoimmunomodulator R837/BMS nanocore using nanoprecipitation followed by electrostatic assembly. Formed R837/BMS@Au8 NP composed of R837, BMS-1, and Au_8_ clusters. Au_8_NC can enhance X-ray absorption at the tumor site to reduce X-ray dose and releases a large number of tumor-associated antigens under X-ray irradiation. With the help of immune adjuvant R837, dendritic cells can effectively process and present tumor-associated antigens to activate effector T cells, meanwhile, a small-molecule PD-L1 inhibitor BMS-1 can block PD-1/PD-L1 pathway to reactivate cytotoxic T lymphocyte, resulting in a strong systemic antitumor immune response that is beneficial for limiting tumor metastasis. According to in vivo and in vitro experiments, radioimmunotherapy based on R837/BMS@Au8 nanoparticles can increase calreticulin expression on of cancer cells, reactive oxygen species generation, and DNA breakage and decrease colony formation. The results revealed that distant tumors were 78.2% inhibited depending on radioimmunotherapy of primary tumors. Therefore, the use of a novel radiosensitizer R837/BMS@Au8 NPs realizes low-dose radiotherapy combined with immunotherapy against advanced cancer.

**Conclusion:**

In conclusion, the multifunctional core–shell R837/BMS@Au8 nanoparticles as a novel radiosensitizer effectively limiting tumor metastasis and decrease X-ray dose to 1 Gy, providing an efective strategy for the construction of nanosystems with radiosensitizing function.

**Supplementary Information:**

The online version contains supplementary material available at 10.1186/s12951-023-02279-2.

## Introduction

Radiotherapy (RT) is one of the mainstays of cancer therapy and has been used for treating 65–75% of patients with solid tumors [1]. However, RT of tumors has two limitations. First, due to the use of high-dose X-rays (50–70 Gy) adjacent normal tissue is damaged [2]. Although radiosensitizers can potentially decrease the toxic effects of X-rays, more efficient radiosensitizers must be developed [3]. Second, conventional RT is often ineffective in preventing tumor metastases, resulting in the death of patients with cancer [4].

Many radiosensitizers have been developed that reduce damage to adjacent normal tissue during RT [5–8]. Additionally, inorganic radiosensitizers with high photostability and low cytotoxicity, such as nanoscintillators, quantum dots, and high-Z metal-based nanomaterials [9–19], have been developed. However, because these are usually large (> 5.5 nm), renal clearance is a challenge, leading to liver damage. Gold nanoclusters (AuNCs) are smaller (1–3 nm) than the kidney filtration threshold (5.5 nm) [20]; and can therefore, achieve efficient renal clearance and reduce liver damage. Xie et al. synthesized an effective glutathione-protected Au_25_NC radiosensitizer that could escape absorption by the reticuloendothelial system during RT [21]. Liang et al. synthesized RGD peptide-modified AuNCs that could target cancer cells with low-dose X-rays [22].

Several AuNCs are promising radiosensitizers [23–25]. However, they can treat local tumors, but not limit tumor metastasis [26–30]. Although radiation therapy induces immunogenic cell death (ICD), in most cases, the antitumor immune responses is not robust enough to control tumor metastases [31, 32]. Therefore, developing a multifunctional composite radiosensitizer to be used to effectively destroy local solid tumors, while inhibiting tumor metastases, is an urgent clinical need.

Immune adjuvants are auxiliary stimulants, such as TLR7 agonists, that strengthen the immune response by promoting antigen-processing and antigen presentation by antigen presenting cells, mainly comprising mature dendritic cells (DCs) [33]. Because cancer cell debris contains tumor-associated antigens (TAAs) post-RT, adding immune adjuvants as auxiliary therapeutic agents improved the antitumor immune response and resulted in synergistic antitumor effects [34].

The use of checkpoint blockade immunotherapy by blocking the PD-1/PD-L1 pathway has attracted great interest [35]. Here, immunosuppressive regulatory T cells (Tregs) are inhibited and cytotoxic T lymphocytes (CTLs) are reactivated by blocking the interaction between PD-1 on CTL surface and PD-L1 on cancer cell surface. Although anti-PD-1 or -PD-L1 antibodies are frequently used in clinical and preclinical studies, they have high cost, low permeability, ~ 30% therapeutic response based on individual variations, and immunogenicity (molecular weight of antibodies is 150,000–160,000). Therefore, small-molecule PD-1/PD-L1 inhibitors, with lower cost and fewer side effects, as well as higher permeability, are considered more suitable for medication [36–38]. We combined photodynamic therapy and immunotherapy based on small-molecule PD-L1 inhibitor BMS-202 to achieve 95% tumor inhibition [39].

**Scheme 1:**
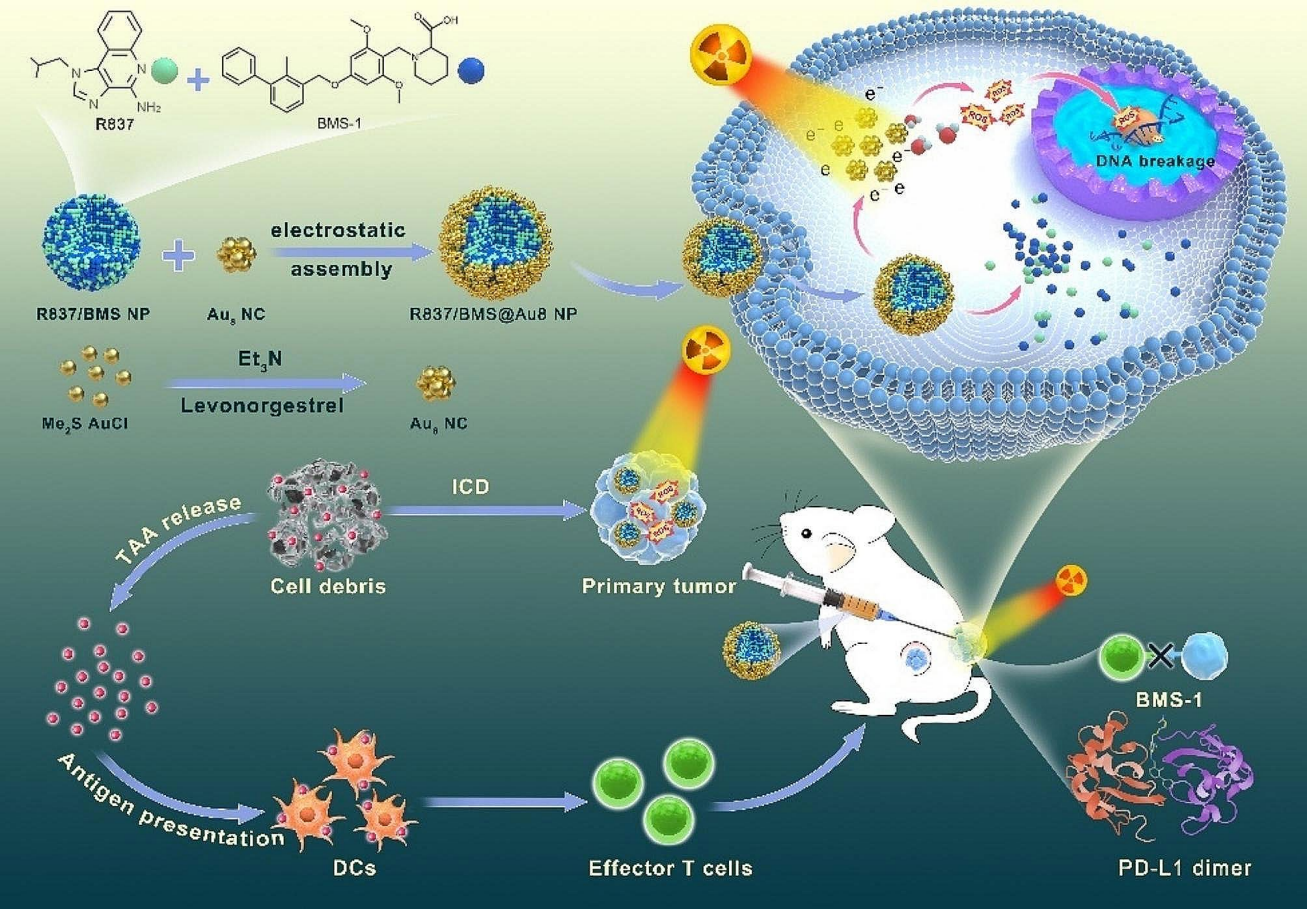
Schematic diagram showing preparation of composite radiosensitizer R837/BMS@Au8 NPs and mechanism of radioimmunotherapy-based treatment of metastatic tumor

In this study, we first synthesized Au_8_ clusters and R837/BMS NPs using a one-pot method and nanoprecipitation, respectively. Then, we assembled Au_8_ clusters on surfaces of bifunctional R837/BMS NPs to form a novel multifunctional composite radiosensitizer for to reducing X-ray dose and limiting tumor metastasis in radioimmunotherapy. Au_8_NCs are effective high-Z radiosensitizers, achieving low-dose X-ray RT against 4T1 tumor, and can effectively be cleared by the kidney. RT based on Au_8_NCs triggered immunogenic cell death (ICD) in 4T1 cells, and the cell debris released TAAs. With the help of immune adjuvant R837, dendritic cells (DCs) can effectively process and present TAAs to activate effector T cells, meanwhile, a small-molecule PD-L1 inhibitor BMS-1 can block PD-1/PD-L1 pathway to reactivate cytotoxic T lymphocyte, resulting in a strong systemic antitumor immune response that is beneficial for limiting tumor metastasis. (Scheme 1). As results, the growth of primary and distant tumors was effectively suppressed depending on radioimmunotherapy of 4T1 tumors based on R837/BMS@Au8 NPs with low-dose X-ray (1 Gy). Inhibition rates were 86.6% and 78.2% for primary and distant tumors, respectively. Therefore, the use of multifunctional and biocompatible composite radiosensitizer R837/BMS@Au8 NPs could realize low-dose RT combined with immunotherapy for enhancing treatment of metastatic tumor.

## Results and discussion

**Fig. 1:**
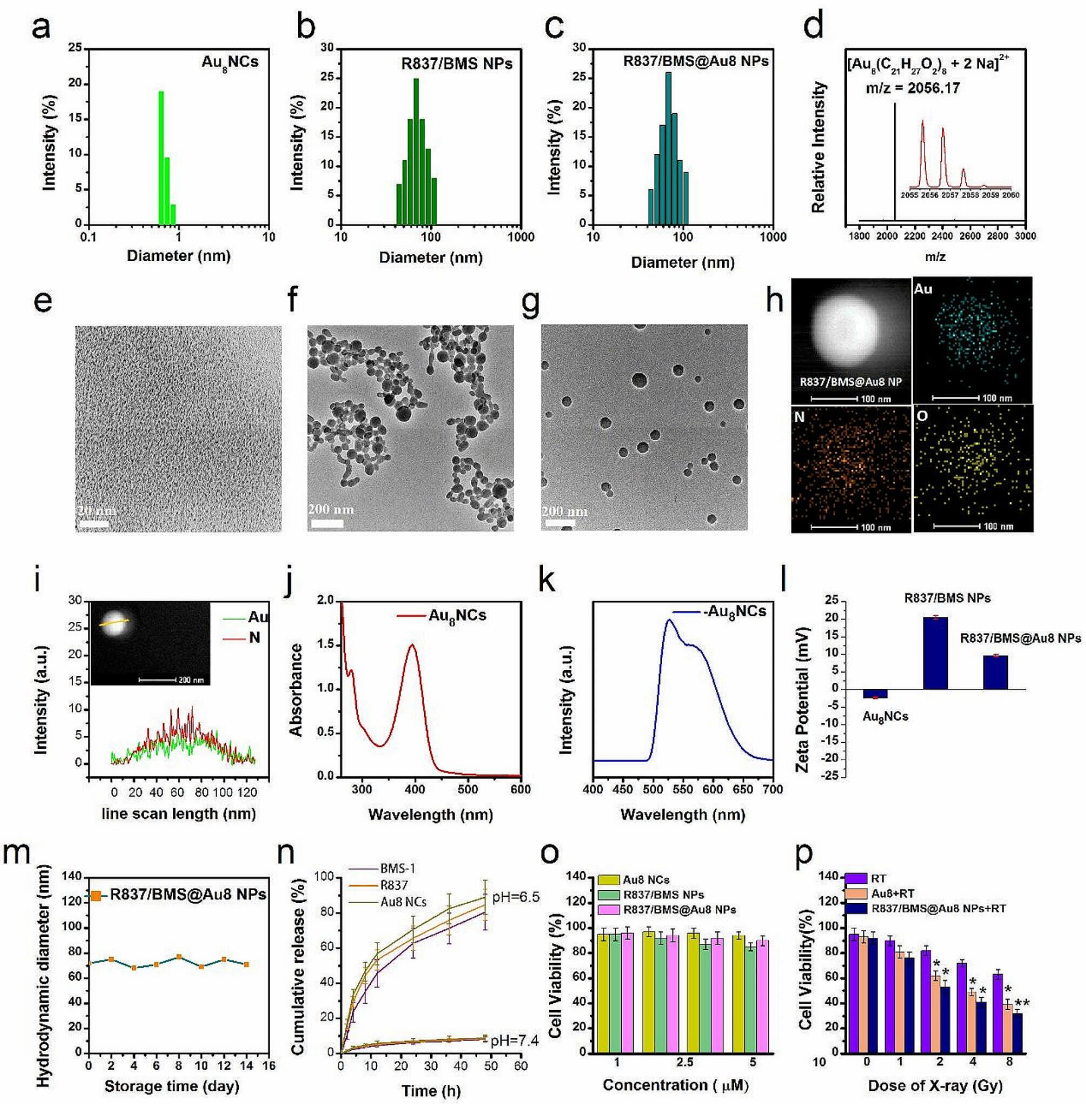
Properties of nanocomposite radiosensitizer. Size distributions of Au_8_ NCs (**a**), R837/BMS NPs (**b**), and R837/BMS@Au8 NPs (**c**). **d** Positive mode ESI-TOF-MS spectrum of Au_8_NCs. Insets: enlarged portion of the ESI-TOF-MS exhibiting isotopic distribution pattern. TEM images of Au_8_NCs (**e**), R837/BMS NPs (**f**), and R837/BMS@Au8 NPs (**g**). **h** Elemental mapping for the R837/BMS@Au8 NP. The element maps show distribution of Au, O, and N. **i** Elemental line scan shows core-shell structure of the R837/BMS@Au8 NP. **j,k** UV–Vis and fluorescence spectra of Au_8_NCs. **l** Zeta potentials of Au_8_NCs, R837/BMS NPs, and R837/BMS@Au8 NPs, n = 3. **m** Storage stability of R837/BMS@Au8 NPs. **n** Release profile of Au_8_NCs, R837, and BMS-1 from R837/BMS@Au8 NPs, n = 3. **o** Cytotoxicity assay of Au_8_NCs, R837/BMS NPs, and R837/BMS@Au8 NPs without radiation, n = 3. **p** Cytotoxicity assay of Au_8_NCs and R837/BMS@Au8 NPs with radiation (RT: radiotherapy). Data are presented as mean ± SD. Statistical significance was determined using the two tailed Student’s t-test. *P < 0.05, **P < 0.01 in comparison with RT group, n = 3

To realize low-dose RT and limit tumor metastasis, a novel nanocomposite radiosensitizer, R837/BMS@Au8 NP composed of R837, BMS-1, and Au_8_ clusters was synthesized using nanoprecipitation followed by electrostatic assembly. Au_8_NCs were prepared as described [5]. Transmission electron microscopy (TEM) and dynamic light scattering (DLS) analyses of Au_8_NCs indicate a size of < 1 nm (Fig. 1a,e), which is smaller than that reported elsewhere [5, 40]. Au_8_NCs display narrower size distribution and better monodispersibility. ESI-TOF-MS analysis showed a main peak of doubly-charged Au_8_NC ion (m/z = 2056.17), corresponding to [Au_8_(C_21_H_27_O_2_)_8_ + 2Na]^2+^ (Fig. 1d). The image in insert shows the isotopic distribution of the main peak. The UV–Vis absorption spectrum shows that the main absorption peaks of Au_8_NCs appear at 225–500 nm (peaks centered at 271, 292, and 388 nm), indicating discrete energy levels (Fig. 1j). The emission spectrum of Au_8_NCs showed intense yellow-green luminescence (Fig. 1k, Fig. S1), with double emission bands at 519 and 578 nm. Luminescence quantum yield was 67.8%, which may be attributed to better monodispersibility and stronger quantum effects of the Au_8_NCs. Then, we synthesized R837/BMS NPs with a mass ratio of 1:2 (R837:BMS-1). TEM analysis showed that R837/BMS NPs are spherical nanoparticles with an average diameter of 68 nm (Fig. 1f). Although R837 and BMS-1 are water insoluble, they contain hydrophilic groups, such as carboxyl and amino groups. When a solution of R837 and BMS-1 in acetone was mixed with a quantity of water, their concentration in the solution exceeded critical thermodynamic solubility. They were in supersaturated states and could co-assemble into an organic composite nanosystem [41, 42].

Finally, R837/BMS@Au8 NPs were synthesized using electrostatic assembly. Au_8_NCs have a negative ζ potential of − 2.38 mV and R837/BMS NPs have a positive ζ potential of 20.3 mV (Fig. 1l), resulting in that Au_8_NCs were assembled on the surface of R837/BMS NPs by electrostatic attraction. However, the formed R837/BMS@Au8 NPs have a positive ζ potential of 12.2 mV, stabilizing the NPs in aqueous medium by electrostatic repulsion. When Au_8_NCs were coated on surface of R837/BMS NPs by electrostatic attraction, the positive ζ potential on R837/BMS NPs was neutralized, resulting in a net positive ζ potential of 12.2 mV of the formed R837/BMS@Au8 NPs. This indicates that the Au_8_NCs layer was not completely dense.

TEM analysis showed that R837/BMS@Au8 NPs are spherical, with an average diameter of 70 nm (Fig. 1g). Element mapping of R837/BMS@Au8 NP confirmed the presence of Au in R837/BMS@Au8 NPs (blue-green fluorescent dots indicate presence of Au) (Fig. 1h). The sizes of R837/BMS NPs and R837/BMS@Au8 NPs determined by DLS were consistent with those determined by TEM (Fig. 1b,c). To verify the Au_8_NCs coating on the surface of R837/BMS NP, we performed an elemental line scan of R837/BMS@Au8 NP (Fig. 1i). These results indicate that there is more Au element than N (in BMS-1 and R837) at the edges of nanoparticles, confirming core-shell structure of the R837/BMS@Au8 NP. The storage experiment displays that R837/BMS@Au8 NPs were stable in phosphate-buffer saline (PBS) (10 µM) for 14 d (Fig. 1m).

In previous work, Liu et al. developed a lipid-based nanocarrier doubly loaded with R837 as a toll-like receptor 7 agonist, and caffeine as an adenosine receptor antagonist to limit the growth of secondary tumors [43]. In a recent study, Au/Ag nanorods were synthesized to inhibit primary and distant metastatic tumor growth [44]. Compared with the two nanostructures mentioned above, our R837/BMS@Au8 NPs have two advantages: first, their 100% drug-loading capacity is beneficial for improving the efficiency of anti-metastatic tumor treatment and overcoming the side effects of carriers; second, Au_8_NCs with ~ 0.7 nm in size are much smaller than the kidney filtration threshold (5.5 nm) and can achieve efficient renal clearance and reduce liver damage.

Therefore, our nanocomposite R837/BMS@Au8 NPs showed good potential as anti metastatic tumor therapeutic agent due to its clear composition, good biocompatibility, suitable granularity, 100% drug loading capacity, and efficient renal clearance. R837/BMS@Au8 NPs are unique among reported antitumor nanomedicines used to radioimmunotherapy of tumors. To assess the bioavailability of R837/BMS@Au8 NPs, the release of BMS-1, R837, and Au_8_NCs from R837/BMS@Au8 NPs was monitored at pH 6.5, which corresponds to the tumor microenvironment (Fig. 1n). Cumulative release curves of BMS-1, R837, and Au_8_NCs from R837/BMS@Au8 NPs were assessed by UV–Vis and dialysis. After 48 h, BMS-1, R837, and Au_8_NCs achieved > 80% release.

For comparison, we performed an additional experiment to evaluate drug leakage in PBS (pH = 7.4). Based on the results of drug release results at pH 6.5 and 7.4, we propose a drug release mechanism: Although BMS-1, R837, and Au_8_NCs are water insoluble, they contain carboxyl, amino, and hydroxyl groups (in ligand of Au_8_NCs), respectively. Therefore, they can be protonated to become water-soluble at pH 6.5, leading to the sustained release of BMS-1, R837, and Au_8_NCs from the R837/BMS@Au8 NPs. Whereas BMS-1, R837, and Au_8_NCs can be deprotonated to maintain hydrophobicity at physiological pH, they are stable and generate a slight release at pH 7.4 due to the effect of stirring during the release process.

To assess the cytotoxicity of Au_8_NCs, R837/BMS NPs, and R837/BMS@Au8 NPs, the CCK-8 test was used to measure the 4T1 cell response. Au_8_NCs, R837/BMS NPs, and R837/BMS@Au8 NPs at concentrations < 10 µM displayed > 80% cell viability, and are therefore, biocompatible at 10 µM (Fig. 1o). The in vitro cytotoxicity of same concentration (10 µM) of Au_8_NCs and R837/BMS@Au8 NPs irradiated using various doses of X-rays was measured by comparing 4T1 cell viability in three cohorts: irradiation only (purple bars); Au_8_NCs + irradiation (brown bars), and R837/BMS@Au8 NPs + irradiation (dark blue bars) (Fig. 1p). Only RT displayed less cytotoxicity. Comparatively, as a radiosensitizer, Au_8_NCs made 4T1 cells more sensitive to radiation, decreasing 4T1 cell viability. Furthermore, 4T1 cell viability sharply decreased when radioimmunotherapy was based on R837/BMS@Au8 NPs, indicating synergy between RT and immunotherapy.

Since the experimental model used in this study was devoid of immune cells and the immune adjuvant R837 and small-molecule PD-L1 inhibitor BMS-1 did not directly kill cancer cells, the cell viability of R837/BMS@Au8 NPs was reduced in comparison to Au_8_NCs only under X-ray irradiation. To explain this phenomenon, we conducted a drug endocytosis experiment. 4T1 cells were incubated with R837/BMS@Au8 NPs or Au_8_NCs with same concentration ( 10 µM) for 24 h. Two sets of cells were collected, precipitated, then dispersed in HNO_3_ solution and digested with H_2_O_2_ under boiling conditions at 200 ℃. ICP-MS was used to determine the Au content of the samples. The results indicated that the Au content in the cells containing Au_8_NC was 63% of that in the cells containing R837/BMS@Au8 NPs (designed 100%). We propose that because Au_8_NC is only ~ 1 nm in size, its amount internalized by cells is less than that of R837/BMS@Au8 NP at same time and concentration, owing to a R837/BMS@Au8 NP (~ 70 nm) containing many Au_8_NCs. Therefore, we conclude that the higher cytotoxicity induced by R837/BMS@Au8 was not related to the function of R837 or BMS-1 but rather to R837/BMS@Au8 NPs, which increased the endocytic amount of Au_8_NCs in cells. The synergistic effect of the three components of R837/BMS@Au8 indirectly kills cancer cells in vitro. As the radiation dose increased, 4T1 cell viability decreased in all three groups. At 1 Gy X-ray, 4T1 cell viability was 43% (< 50%). Hereafter, 1 Gy X-rays are used as radiation source for in vivo and in vitro antitumor experiments.

**Fig. 2:**
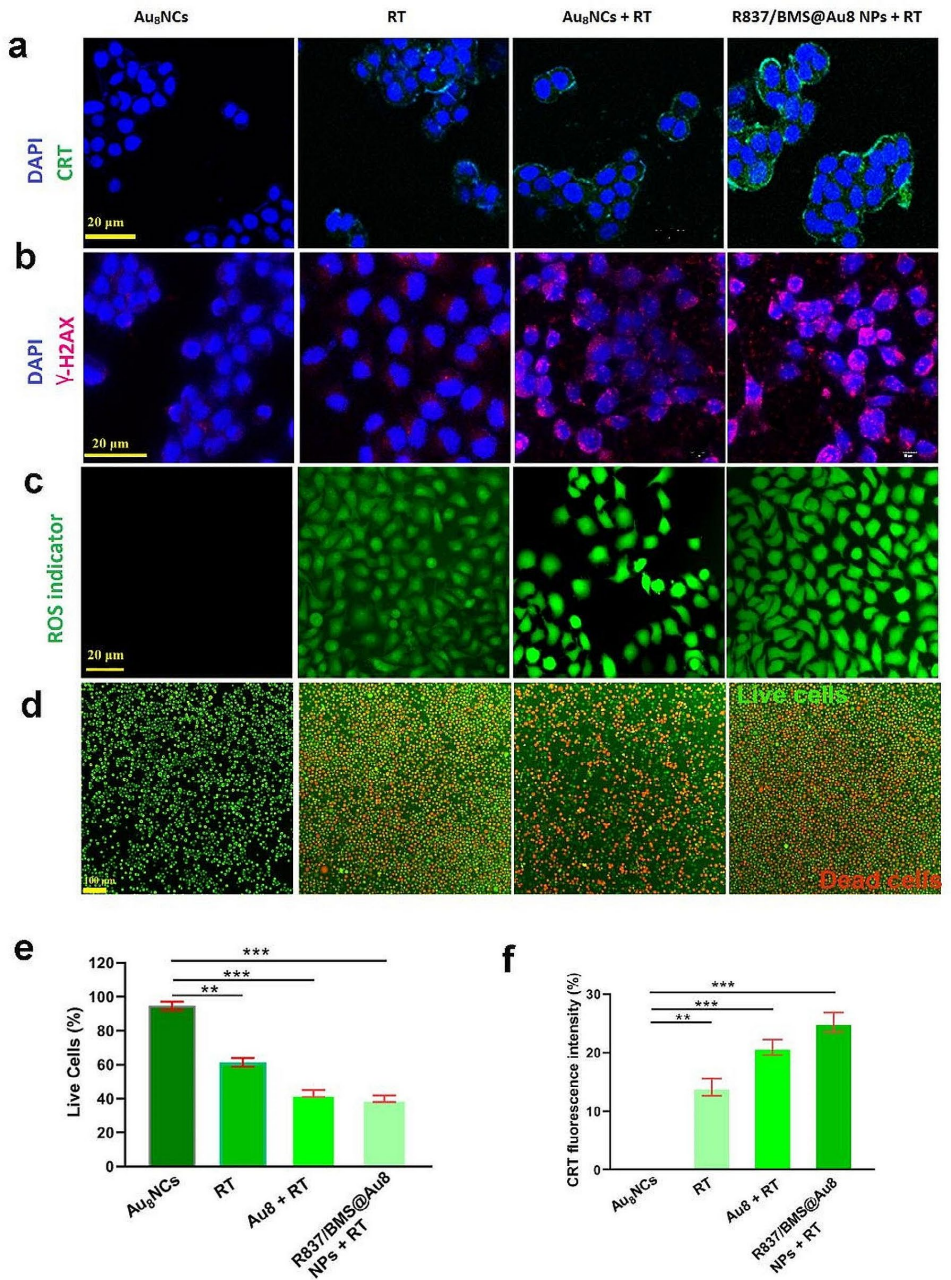
In vitro radiosensitization effects. **a** CLSM images of calreticulin expression on 4T1 cells after receiving different treatments. **b** CLSM images of γ-H2AX stained 4T1 cells after different treatments. **c** CLSM images of ROS generation in 4T1 cells after different treatments. **d** Live/dead imaging of 4T1 cells after receiving different treatments. Green: live; red: dead. **e** The amount of Live/dead cells after various treatments. **f** The amount of Calreticulin expression on 4T1 cells after various treatments. Data are presented as mean ± SD. Statistical significance was determined using the two tailed Student’s t-test. **P < 0.01, ***P < 0.001 in comparison with Au_8_NC group, n = 3

Au nanomaterials have excellent biocompatibility and ability to concentrate local radiation [45]; therefore, the role of the prepared Au_8_NCs and R837/BMS@Au8 NPs as radiosensitizers were evaluated in vitro *.* Briefly, 4T1 cells received treatments including: Au_8_NCs, RT, Au_8_NCs + RT, and R837/BMS@Au8 NPs + RT, to evaluate calreticulin (CRT) expression, reactive oxygen species (ROS) generation, DNA breakage, and live/dead cells.

The anti-immune response during RT has attracted great interest recently. RT-induced TAAs were reported to promote the antitumor immune response [46]. In cancer cells, RT induces ICD, which is characterized by high CRT expression (“eat me” signal) on the surface of apoptotic cancer cells. Therefore, CRT expression was evaluated on the surface of 4T1 cells treated with Au_8_NCs, 1 Gy RT, Au_8_NCs + 1 Gy RT, and R837/BMS@Au8 NPs + 1 Gy RT (Fig. 2a,f). The treated cells were stained with FITC-conjugated anti-CRT antibody and imaged using a confocal microscope. In the Au_8_NCs + 1 Gy RT and R837/BMS@Au8 NPs + 1 Gy RT groups, higher CRT expression was visible on the surface of 4T1 cancer cells than Au_8_NCs and RT groups. R837/BMS@Au8 induces a higher expression of calreticulin (CRT), which is not related to function of R837 or BMS-1. However, because R837/BMS@Au8 NPs increase the endocytic amount of Au_8_NCs in cancer cells, resulting in more severe immunogenic cell death.

Studies have shown that using Au NPs as radiosensitizer causes water radiolysis in cancer cells, generating excess ROS which interacts with biomolecules, leading to breakage of DNA double strand, cell apoptosis, etc. [47, 48]. 4T1 cells treated with Au_8_NCs or R837/BMS@Au8 NPs were incubated with DCFH-DA (a probe for ROS) and irradiated with 1 Gy X-rays. ROS produced was detected using a fluorescence microscope (Fig. 2c). Au_8_ NCs + RT and R837/BMS/Au8 NPs + RT displayed stronger green fluorescence signals than RT or Au_8_NCs.

RT kills cancer cells through ROS-induced DNA damage. The degree of DNA damage in 4T1 cells after X-ray irradiation was detected by the immunofluorescence staining of γ-H2AX, which is a marker of DNA damage. γ-H2AX foci were absent in the nuclei of the Au_8_NC group and fewer γ-H2AX foci were present in the nuclei of the RT group (Fig. 2b). By contrast, higher densities of γ-H2AX foci were found in Au_8_NCs + RT and R837/BMS@Au8 NPs + RT groups, demonstrating Au_8_NC-induced DNA breakage. These results are consistent with those for the ROS generation test.

To assess the therapeutic effect of our nanocomposite radiosensitizer R837/BMS@Au8 NPs, the cells were stained with Calcein-AM/PI. Live and dead cell imaging showed that 4T1 cells in the PBS group had the highest survival rates (Fig. 2d). Comparatively, 4T1 cells in other groups showed different degrees of apoptosis. Apoptotic cell number was highest in the group that received radioimmunotherapy based on R837/BMS@Au8 NPs (Fig. 2e).

**Fig. 3:**
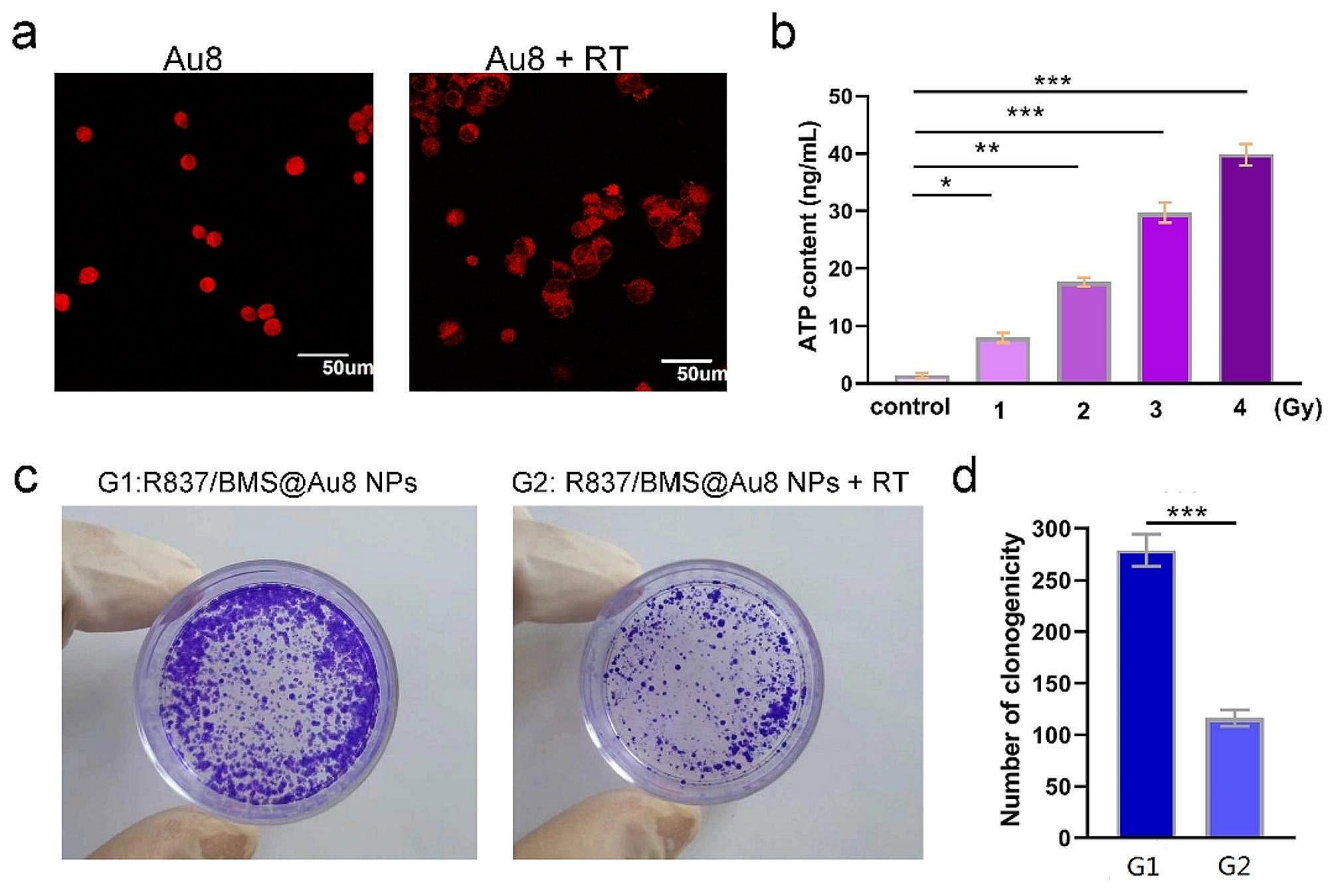
**a** Immunofluorescence checking was shown and verified the specific localization of HMGB1 before and after irradiation. **b** Relationship between radiotherapy dose and ATP concentration released by tumor cells. **c** Representative images of the colony formation assay of 4T1 cells in R837/BMS@Au8 NP and R837/BMS@Au8 NPs + RT groups. **d** Numbers of clonogenity in the two groups. Data are presented as mean ± SD. Statistical significance was determined using the two tailed Student’s t-test. *P < 0.05, **P < 0.01, ***P < 0.001 in comparison with control or G1 group, n = 3

HMGB1 is a DNA-binding protein released during radiotherapy. Using CLSM, we investigated the cell-specific localization of HMGB1 in 4T1 cells with or without 1 Gy X-ray treatment. As shown in Fig. 3a, HMGB1 was released from the nucleus into the cytoplasm. The results suggested that compared with the non-irradiated groups, the 1 Gy-irradiated cells had an increased distribution of HMGB1 protein in the cytoplasm.

To verify that Au_8_NCs can respond in the proximity of tumor tissues, we assessed the radiation-induced changes in ATP concentration in tumor cells (Fig. 3b). With increasing RT dosage, the concentration of ATP in the tumor cell medium gradually increased, thereby facilitating the response of Au_8_NCs in the tumor tissue.

To examine the in vitro radiosensitizing effects of Au_8_NCs during 14 d, the colony formation assay was performed (Fig. 3c,d). In the control group (only R837/BMS@Au8 NPs), the colonies were densely packed, indicating that R837/BMS@Au8 NPs exhibit outstanding biocompatibility at the experimental concentration and had no observed influence on cell proliferation. Compared to the R837/BMS@Au8 NP group in the absence of X-ray irradiation, the surviving fractions of the group treated with R837/BMS@Au8 NPs + RT were obviously lower.

Thus, R837/BMS@Au8 NPs can effectively induce ROS generation, DNA breakage, and CRT expression resulting in increased cell apoptosis and decreased cancer cell colony formation under X-ray irradiation. R837/BMS@Au8 NPs is an effective radiosensitizer and can be applied in next generation RT.

**Fig. 4:**
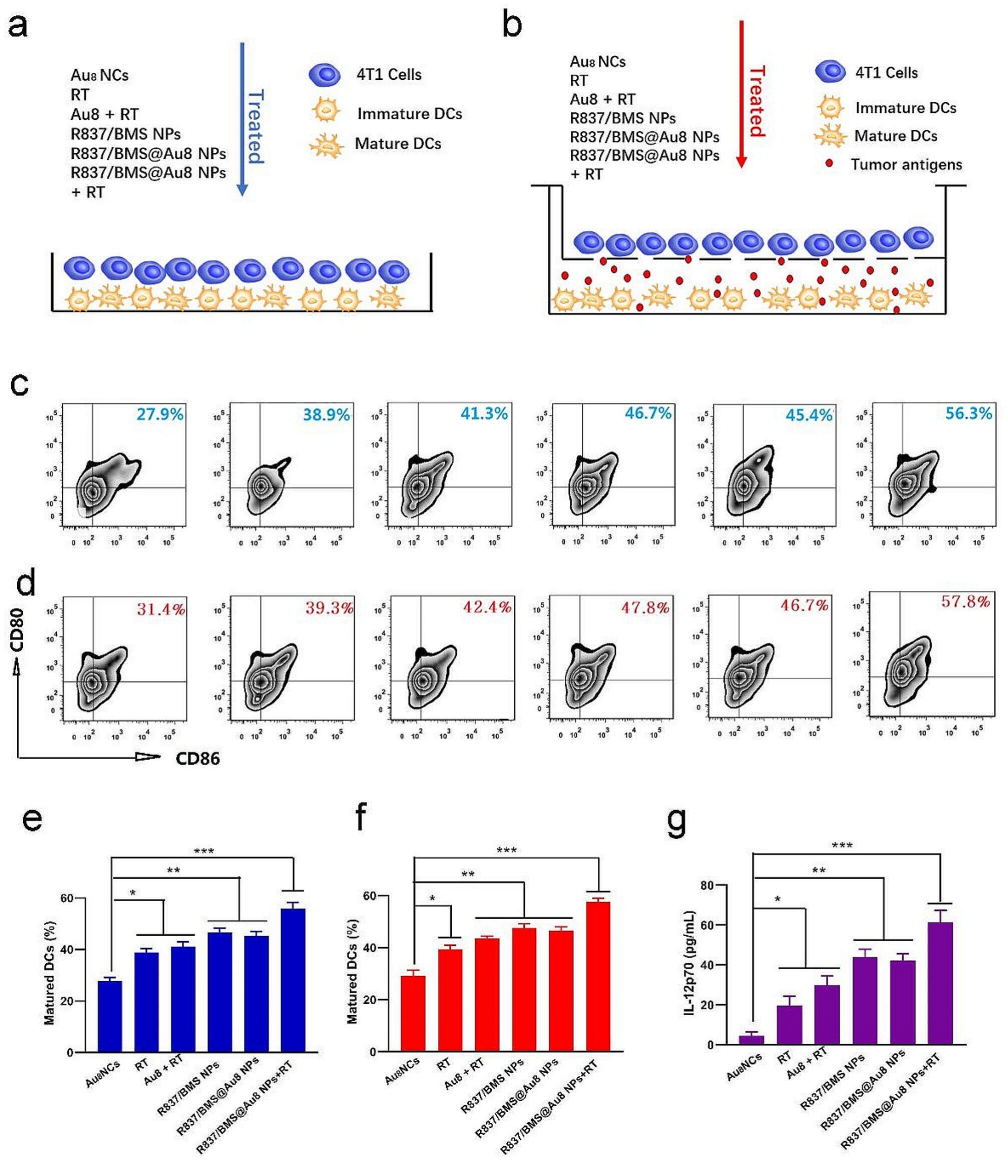
In vitro DC maturation. **a, b** Schemes showing the design of two DC maturation experiments in vitro. **c, e** The amount of mature DCs after various treatments in vitro coculture system. **d,f** The amount of mature DCs after various treatments in Transwell system. **g** IL-12p70 secreted in suspensions of coculture system **b**. Data are presented as mean ± SD. Statistical significance was determined using one-way ANOVA test. *P < 0.05, **P < 0.01, ***P < 0.001, n = 3

Immature DCs engulf TAAs and process them into peptides, which are presented to the major histocompatibility complex (MHC). The MHC–TAA complex on DC surface is recognized by T cell receptors to activate T cells depending on the maturation of DC [49, 50]. This initiates the antitumor immune response. We first investigated in vitro DC maturation stimulated by treatment strategies. Immature DCs from the bone marrow were stimulated with granulocyte macrophage colony stimulating factor and then given various treatments in a DC/4T1 coculture system. In direct mixing of DCs and 4T1 cells, coculturing of dual cells was followed by treatments including PBS, RT, Au_8_NCs + RT, R837/BMS NPs, R837/BMS@Au8 NPs, and R837/BMS@Au8 NPs + RT (Fig. 4a). Mature DCs were confirmed by dual staining for CD86 and CD80, which are markers of mature DCs (Fig. 4c,e). The percentage of mature DCs (CD80^+^ CD86^+^) in the treatment group with R837/BMS@Au8 NPs + RT was the highest (up to 56.3%) compared with those in other groups. To confirm this result, a Transwell system was used with upper and lower chambers containing 4T1 cells and DCs, respectively, followed by different treatments (Fig. 4b). The frequency of mature DCs (CD80^+^ CD86^+^) in the R837/BMS@Au8 NPs + RT treatment group was also the highest (57.8%) (Fig. 4d,f). Two sets of experimental results proved that the most evident maturation of DCs was observed in the R837/BMS@Au8 NPs + RT group, indicating that radioimmunotherapy based on R837/BMS@Au8 NPs can efficiently stimulate DC maturation. Studies have reported that tumor cell RT induces TAA generation, and R837 acts as an immune adjuvant to stimulate DC maturation. Furthermore, the levels of interleukin 12 (IL-12p70) in the medium of the Transwell system after treatments were measured via enzyme-linked immunosorbent assay (ELISA). IL-12p70 is a cytokine that is secreted by DCs. The results showed that the changing trend of IL-12p70 is similar to that of mature DCs in both coculturing modes (Fig. 4g).

**Fig. 5:**
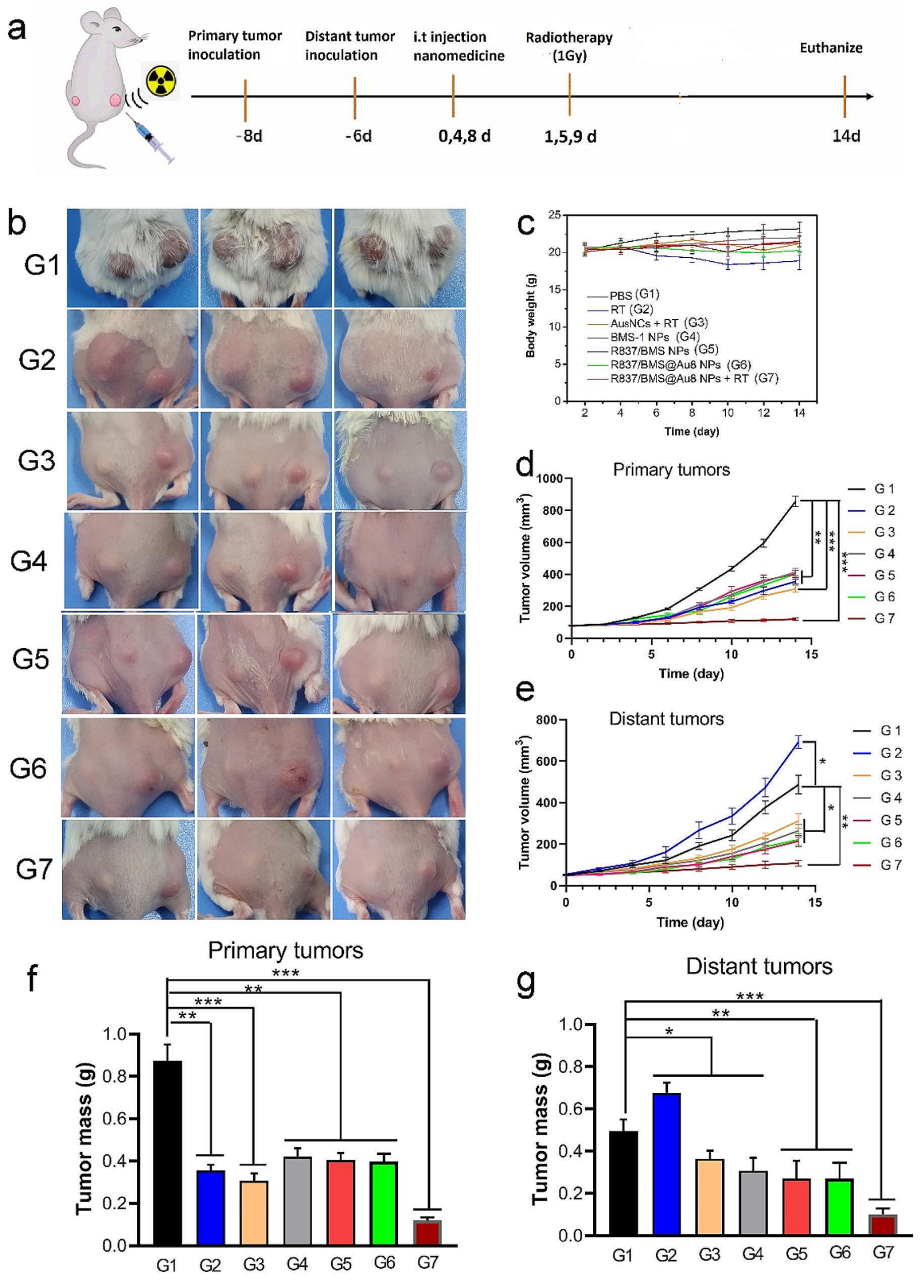
In vivo antitumor test. **a** Treatment schedule. **b** Photographs of euthanized mice after 14 d of treatment. **c** Changes of body weights of mice during tumor treatments. **d,e** Growth curves of primary and distant tumor volumes. **f,g** Weights of primary and distant tumors after tumor treatments. Data are presented as mean ± SD. Statistical significance was determined using one-way ANOVA test. *P < 0.05, **P < 0.01, ***P < 0.001, n = 5

The in vitro effects of radioimmunotherapy based on R837/BMS@Au8 NPs + RT inspired us to investigate the effects of radioimmunotherapy of tumor in vivo. Therefore, 4T1 cells were inoculated into the right flanks of the mice as primary tumors. After 2 d, distant tumors, as mimic metastasis tumors, were inoculated into the left flanks of the same mice (Fig. 5a). Simultaneously, 4T1 cells were injected into the same mice through the tail vein to form pulmonary nodular tumors. When primary and distant tumors reached approximately 80 and 50 mm^3^, respectively, the mice were randomly divided into seven groups (n = 5): (1) PBS; (2) RT only; (3) Au_8_NCs + RT; (4) intratumoral (i.t.) injection of BMS-1 NPs; (5) i.t. injection of R837/BMS NPs; (6) i.t. injection of R837/BMS@Au8 NCs; and (7) i.t. injection of R837/BMS@Au8 NCs + RT. Au_8_ NCs, BMS-1 NPs (Fig S2), R837/BMS NPs, and R837/BMS@Au8 NPs, were locally injected into tumors in the corresponding groups at doses of 4 mg/kg for Au_8_NCs, 1 mg/kg for BMS-1, and 0.5 mg/kg for R837 on day 0, 4, 8 after treatment, respectively. After 24 h, relational primary tumors were radiated with 1 Gy X-ray on day 1, 5, 9 after treatment, respectively. To avoid cytokine storms, i.t. injection is used for radioimmunotherapy of tumors. Then, tumor size on both sides was monitored every other day. Primary tumors in groups with X-ray radiation, BMS-1 NPs, R837/BMS NPs, and R837/BMS@Au8 NPs showed slower growth compared with those in the PBS group (Fig. 5b, d). The primary tumor treatments in RT and Au_8_NCs + RT groups were found to be more effective than the above four groups including PBS, BMS-1 NPs, R837/BMS NPs, and R837/BMS@Au8 NPs groups. R837/BMS@Au8 NPs + RT was most effective in inhibiting primary tumor growth. Thus, R837/BMS@Au8 NPs could improve the efficacy of RT by promoting absorption of X-rays through Au_8_NCs, strengthening antitumor immunity through R837, and blocking the PD-1/PD-L1 pathway through BMS-1.

**Fig. 6:**
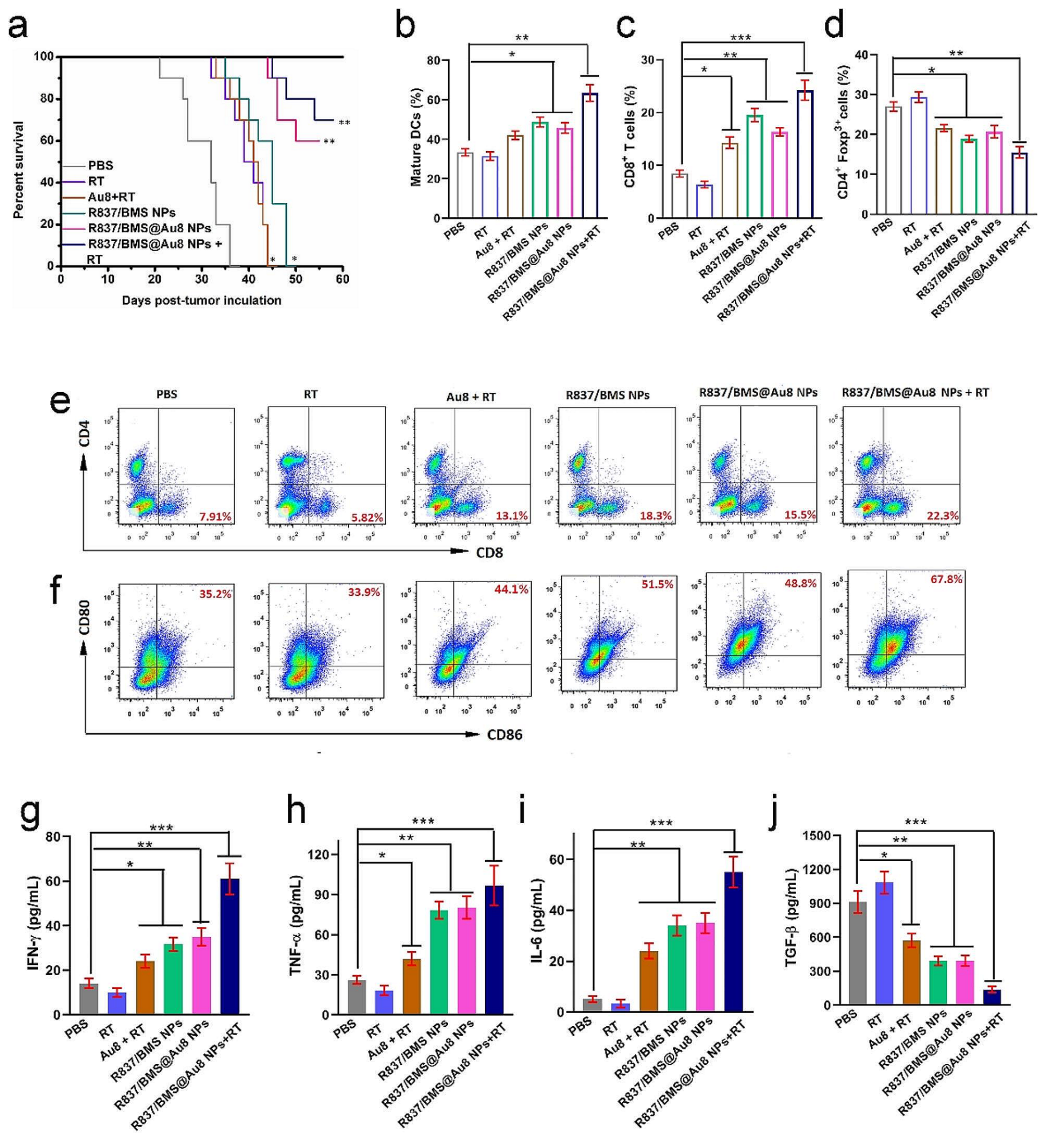
Mechanism of anti metastatic tumor. **a** Survival rates of mice after various treatments (n = 10). **c,e** Infiltration of CD8^+^cells into distant tumors. **d** Infiltration of CD4^+^Foxp3^+^cells into distant tumors. **b,f** DC maturation rates from the lymph nodes in bilateral 4T1 tumor mice. **g–j** Serum IFN-γ, TNF-α, IL-6, and TGF-β levels. Data are presented as mean ± SD. Statistical significance was determined using one-way ANOVA test. *P < 0.05, **P < 0.01, ***P < 0.001, n = 3

The growth of distant tumors (as simulated metastatic tumors without direct treatment) was inhibited possibly due to the activation of antitumor immunity. The distant tumors of mice with primary tumors irradiated with 1 Gy displayed rapid growth (Fig. 5b,e), because RT alone could not activate the antitumor immune response in mice. The distant tumors of mice with primary tumors injected with BMS-1 NPs, R837/BMS NPs or R837/BMS@Au8 NPs had slower growth than those in the RT only group, demonstrating the importance of R837 in inducing antitumor immunity and BMS-1 in blocking the PD-1/PD-L1 pathway. In RT + Au_8_NC group, the distant tumors had comparable growth with tumors in the above three groups, indicating that radioimmunotherapy based on Au_8_NCs induces systemic antitumor immunity. Notably, 3/5 distant tumors with corresponding primary tumors treated with injection of R837/BMS@Au8 NPs + RT shrank. Thus, radioimmunotherapy based on R837/BMS@Au8 NPs decreases the dose of X-ray and initiates strong systemic antitumor immunity to limit tumor metastasis. At the end of the experiment, all primary and distant tumors were dissected from the mice and weighed. The inhibition rates of primary and distant tumors were 86.6% and 78.2%, respectively (Fig. 5f,g). Furthermore, slight changes in mice weight were observed during treatment (Fig. 5c), suggesting low systemic toxicity of various therapies. Additionally, radioimmunotherapy based on R837/BMS@Au8 NPs enhanced the survival time of tumor-bearing mice, compared with mice in the other five groups. Survival period of 70% mice in the R837/BMS@Au8 NPs + RT group was > 60 d, whereas the survival time of tumor-bearing mice was 36 d in the PBS group (Fig. 6a). ICP-MS analysis confirmed that Au_8_NCs were eliminated in the urine of tumor-bearing mice within 9 d.

We then examined the mechanisms of antitumor metastasis during radioimmunotherapy based on R837/BMS@Au8 NPs. PD-L1 blockade could prevent immune evasion of cancer cells to combat tumors by reactivating CTLs and inhibiting immune-suppressive Tregs [39]. CTLs (CD3^+^CD4^−^CD8^+^) directly kill cancer cells by releasing cytotoxins, such as IFN-γ, granzymes, granulysin, and perforin [51, 52]. Distant tumors were removed from 4T1 tumor-bearing mice. CD8^+^ CTLs (CD3^+^CD4^−^CD8^+^) were isolated for analyzing their percentages in different treatment groups by flow cytometry on day 6 after treatment. Compared to the control groups (7.91% for PBS group and 5.82% for RT group), Au8 + RT, R837/BMS NP, and R837/BMS@Au8 NP groups had 13.1%, 18.3%, and 15.5% of CD8^+^ T cells, respectively (Fig. 6c,e). Notably, for the R837/BMS@Au8 NPs + RT group, the frequency of CD8^+^ CTLs was the highest at 23.3%. Conversely, the percentage of Tregs (CD4^+^Foxp3^+^ T cells) in the R837/BMS@Au8 NPs + RT group was the lowest in distant tumors (Fig. 6d).

On day 5 after treatment, we examined in vivo DC maturation in inguinal lymph nodes, and evaluated levels of interferon-γ (IFN-γ), tumor necrosis factor α (TNF-α), IL-6, and TGF-β by ELISA in mouse sera. The frequency of mature DCs (CD11c^+^CD80^+^CD86^+^) was highest in the R837/BMS@Au8 + RT group (67.8%) compared with PBS, RT, Au_8_NCs + RT, R837/BMS NPs, R837/BMS@Au8 NPs groups (Fig. 6b,f). We measured the levels of IFN-γ, TNF-α, IL-6, and TGF-β in the sera of mice from different treatment groups on day 4 after treatment. Cytokines are important markers of innate and adaptive immunity. IL-6 is proinflammatory; IFN-γ and TNF-α are key markers of cellular immunity and play important roles in tumor immunotherapy; and TGF-β is immunosuppressive. Levels of TNF-α, IL-6, and IFN-γ were upregulated, whereas that of TGF-β was significantly decreased in the R837/BMS@Au8 NPs + RT group (Fig. 6g–j). Taken together, radioimmunotherapy based on R837/BMS@Au8 NPs strengthened in vivo antitumor immunity due to the adjuvant function of R837, TAA release after improved RT by Au_8_NCs, and blockade of PD-L1 function by BMS-1.

**Fig. 7:**
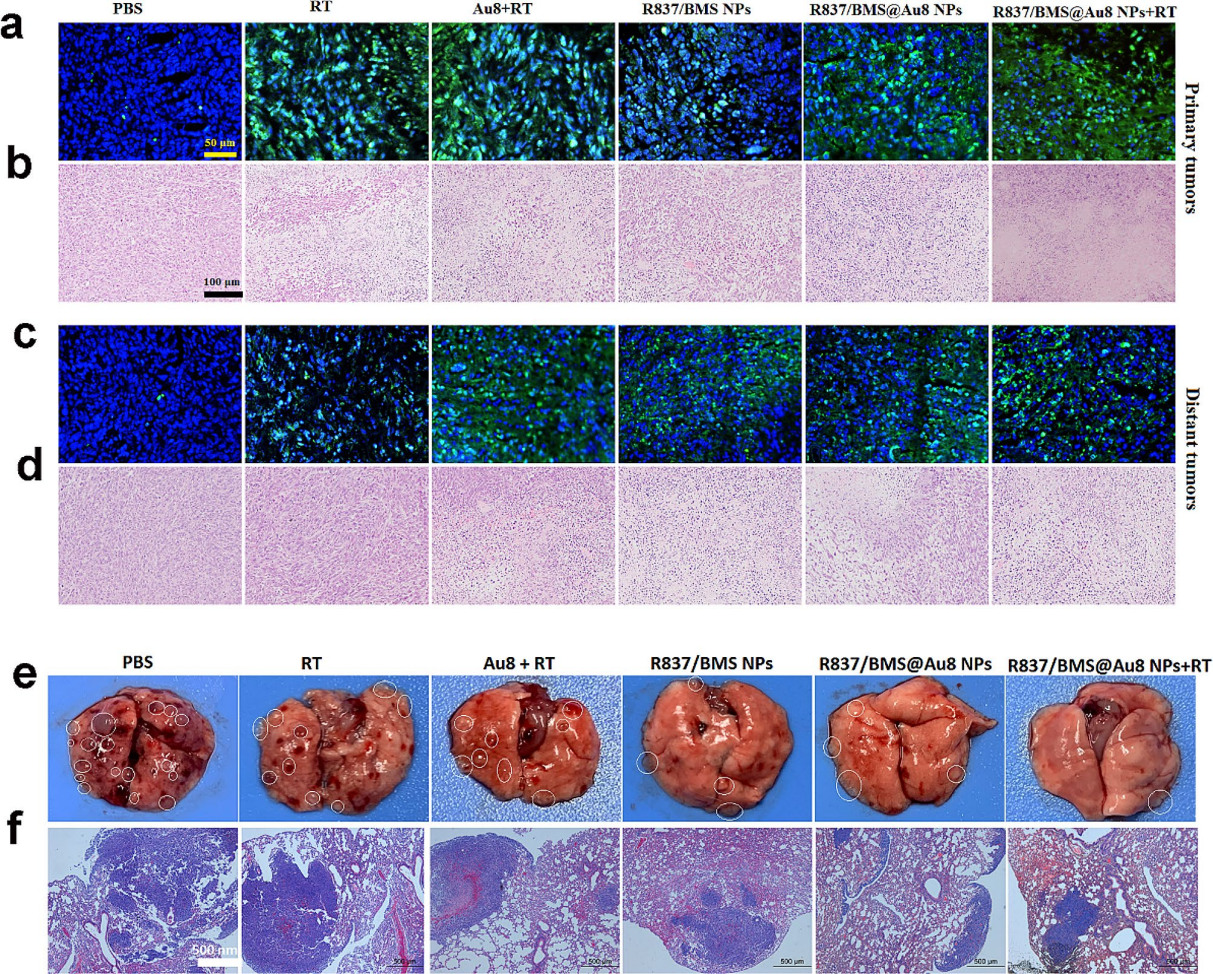
**a,b** TUNEL and H&E images of primary tumors after different treatments. **c,d** TUNEL and H&E images of distant tumors after different treatment. **e,f** Representative images showing the gross appearance of tumor nodules in the lungs and lung sections stained with H&E.

To evaluate 4T1 tumor cell damage and apoptosis in vivo, sections of primary and distant tumors from different therapeutic groups were stained with hematoxylin and eosin (H&E). Different degrees of cellular damage were seen in RT, Au8 + RT, R837/BMS NP, and R837/BMS@Au8 NP groups. The most serious damage (cell shrinkage and chromatin condensation) was observed in the R837/BMS@Au8 NPs + RT group (Fig. 7b). The terminal deoxynucleotidyl transferase-mediated dUTP nick-end labeling (TUNEL) test was used to detect apoptotic cells. RT, Au8 + RT, R837/BMS NP, and R837/BMS@Au8 NP groups showed evident cell apoptosis, whereas the R837/BMS@Au8 NPs + RT group displayed the most evident apoptotic cells (Fig. 7a). Images for H&E staining and TUNEL showed similar analysis results for untreated distant tumor sections (Fig. 7c,d).

To assess the effect of treatments limiting the formation of metastatic tumor nodules after tail vein injection of 4T1 cells during therapy, the lung was isolated from all groups at the end of the in vivo antitumor experiment. Many metastatic tumor nodules were found on the surface of the lung in PBS, RT, and Au_8_NCs + RT groups. By contrast, fewer nodules were present in groups R837/BMS NP and R837/BMS@Au8 NP. In group R837/BMS@Au8 NPs + RT, only one metastatic nodule was found in the lung (Fig. 7e). Lungs with metastatic nodules in different treatment groups were sectioned and stained with H&E to examine the metastatic area (Fig. 7f). The trend of change in tumor nodule number correlated with trend of change in lung metastasis area. The two sets of results confirmed that RT using R837/BMS@Au8 NPs as radiosensitizer could effectively limit lung tumor nodules, possibly due to increased antitumor immune response and reduced X-ray dose.

The systemic toxicity of nanomedicines is a key index for their clinical transformation. H&E staining was performed at the end of the antitumor experiment to evaluate systemic toxicities of the radiosensitizer R837/BMS@Au8 NPs in vivo. Histological analysis revealed no damage to the heart, liver, spleen, lung, and kidney of mice in all treatment groups (Fig. S3).

## Conclusion

By using a novel nanocomposite radiosensitizer, R837/BMS@Au8 NPs, we combined RT with immunotherapy to achieve promising systemic antitumor therapeutic effects. The treatment inhibited local tumor growth and tumor metastases and increased treatment safety. Au_8_NCs absorbed more photons to decrease the dose of X-rays in primary tumors, exhibiting higher treatment safety and TAA release. The adjuvant R837 assisted in processing and presenting TAAs to stimulate the maturation of DCs, which reactivated T cells. The small-molecule inhibitor BMS-1 blocked the PD-1/PD-L1 pathway by forming the PD-L1 dimer, resulting in the reactivation of T cells. Therefore, in vitro radioimmunotherapy based on R837/BMS@Au8 NPs generated more ROS, induced severe DNA breakage, decreased cancer cell colony formation, and secreted high levels of CRT, resulting in cancer cell apoptosis. Tumor inhibition rates for primary and distant tumors were 86.6% and 78.2%, respectively, with R837/BMS@Au8 NPs as an antitumor agent. This study not only provides a novel multifunctional nanocomposite radiosensitizer R837/BMS@Au8 NPs, but also contributes to an effective antitumor treatment strategy.

## Experimental section

### Preparation of R837/BMS@Au8 NPs

Au_8_ NCs were synthesized as described [40] with slight modification. Briefly, 25 µM levonorgestrel in mixed solvent (DCM/CH_3_CN = 1:1) was prepared with magnetic stirring. Then, 25 µL of NEt_3_ was added to 6 mL of levonorgestrel solution. Next, 7.4 mg of Me_2_SAuCl was added to the mixed solution under magnetic stirring, yielding a yellow-green transparent solution. The solution was stirred for 5 min and allowed to evaporate slowly in the dark at room temperature for 2–3 d, to yield a yellow-green powder. R837/BMS nanocores were prepared using a reprecipitation method. First, 400 µL (1 mg/mL) of BMS-1 in acetone and 400 µL (0.5 mg/mL) of R837 in acetone were mixed. Then 800 µL of the mixed solution was drip-added to 5 mL Milli-Q water under magnetic stirring at room temperature. Stirring for 45 min removed acetone from the solution.

To assemble Au_8_NCs on the R837/BMS nanocore, 1.62 mg Au_8_NC was added to 1 ml of deionized water, and the mixture was subjected to ultrasound for 2 h to reach a transparent green solution. Subsequently, 1 ml green solution was slowly added to 2 ml of the R837/BMS NP aqueous dispersion to form R837/BMS@Au8 NPs through electrostatic attraction. The obtained R837/BMS@Au8 NPs had ~ 0.1 mM concentration of all three components (Au_8_NCs, BMS-1 and R837). Finally, R837/BMS@Au8 NPs were purified by dialysis to remove unreactive substances (molecular weight cutoff: 12,000 Da).

### In vitro DC maturation

Two culture systems were used to study DC maturation. (1) DCs and 4T1 cells were co-incubated for 48 h and treated with the following: PBS, X-ray (1 Gy), Au_8_NCs (40 µg/mL) + X-ray (1 Gy), R837/BMS NPs (5 µg/mL for R837 and 10 µg/mL for BMS-1), R837/BMS@Au8 NPs (5 µg/mL for R837, 10 µg/mL for BMS-1, and 40 µg/mL for Au_8_ NCs), and R837/BMS@Au8 NPs + X-ray (1 Gy). After 48-h incubation, the cells were collected and stained with fluorescein-labeled anti-CD11c, anti-CD80, and anti-CD86 antibodies to identify mature DCs via flow cytometry. (2) DCs and 4T1 cells were introduced into the lower and upper chambers of the Transwell coculture system, respectively. Only 4T1 cells in the upper chamber received treatment. DCs were collected and stained with fluorescein-labeled anti-CD11c, anti-CD80, and anti-CD86 antibodies for flow cytometry. Cell culture supernatants were collected to measure the proinflammatory cytokine IL-12p70 secreted by DCs using enzyme-linked immunosorbent assay (ELISA).

### Antitumor study in vivo

All animal experiments were performed and approved at the Institute of Radiation Medicine (Tianjin, China) and adhered to the guidelines of the Committee for Research and Animal Ethics. Bilateral tumor models were established in BALB/c mice by subcutaneously injecting 4T1 cells (1 × 10^5^ and 1 × 10^4^) into the right and left flanks, respectively, to form primary tumors (~ 80 mm^3^) and distant tumors (~ 50 mm^3^). Then, 4T1 tumor-bearing mice were randomly divided into seven groups (n = 5): (1) PBS, (2) RT (1 Gy X-ray), (3) Au8 + RT (1 Gy X-ray), (4) BMS-1 NPs, (5) R837/BMS NPs, (6) R837/BMS@Au8 NPs, and (7) R837/BMS@Au8 NPs + RT (1 Gy X-ray). Au_8_ NCs, BMS-1 NPs, R837/BMS NPs, and R837/BMS@Au8 NPs, were locally injected into tumors in the corresponding groups at doses of 4 mg/kg for Au_8_NCs, 1 mg/kg for BMS-1, and 0.5 mg/kg for R837 on day 0, 4, 8 after treatment, respectively. After 24 h, relational primary tumors were radiated with 1 Gy X-ray on day 1, 5, 9 after treatment, respectively. To avoid cytokine storms, i.t. injection is used for radioimmunotherapy of tumors. Tumor volumes and body weights of mice were measured every other day for 14 d and the tumor volume V was calculated according to equation: V = L × W^2^ / 2. At the end of the experiment, tested mice were euthanized and photographed, the tumors were resected and weighted, and lungs were removed for counting tumor nodules.

### Statistics analysis

All data were presented as mean ± standard deviation (SD) of at least three independent experiments. The GraphPad Prism 8.0 software was used for data analysis. Statistical analysis was performed using the two tailed Stude’s t-test for two groups and one-way analysis of variance (ANOVA) for more than three groups, followed by Tukey’s post-hoc test for multiple comparisons. Statistically significant differences were indicated by *P < 0.05, **P < 0.01, and ***P < 0.001.

### Other experiments

See full experimental details in the Electronic Supplementary Information.

### Electronic supplementary material

Below is the link to the electronic supplementary material.

Supplementary Material 1

## Data Availability

Additional file is available online.
