# Supplementary material for: Assembling Au8 clusters on surfaces of bifunctional nanoimmunomodulators for synergistically enhanced low dose radiotherapy of metastatic tumor
Source: J Nanobiotechnology. 2024 Jan 5;22:20. doi: 10.1186/s12951-023-02279-2 (PMC10768385; doi:10.1186/s12951-023-02279-2)
Supplement: Supplementary file 1 — Supplementary Material 1 [file 12951_2023_2279_MOESM1_ESM.docx]

Additional file

for

**Assembling Au_8_ clusters on surfaces of bifunctional nanoimmunomodulators for synergistically enhanced low dose radiotherapy of** **metastatic tumor**

Rui Zhang^1^^†*^, Mengchao Jia^1†^, Hongying Lv^2^, Mengxuan Li^1^, Guanwen Ding^1^, Ge Cheng^1^, Juan Li^1*^

**Experimental**

**Materials and reagents**

Levonorgestrel was purchased from Hubei Goto Biopharm Co., Ltd. 4',6-diamidino-2-phenylindole (DAPI) was purchased from Sigma-Aldrich. 4T1 cells and RPMI-1640 were purchased from the Cell Bank of Chinese Academy of Sciences (Shanghai, China). Cell Counting Kit-8 (CCK-8), Immunol Staining Fix Solution, crystal violet staining solution were purchased from Beyotime Biotechnology Co., Ltd. LIVE/DEAD™ Cell Imaging Kit, Highly Cross-Adsorbed Secondary Antibody Alexa Fluor™ 647 goat anti-rabbit IgG (H+L), and γ-H2AX antibody were purchased from Thermo Fisher Scientific. BALB/c mice (5-week-old females) were obtained from Beijing Vital River Laboratory Animal Technology Co., Ltd.

**Cytotoxicity analysis**

4T1 cells were seeded into 96-well plates at a density of approximately 2 × 10^4^ cells per well and cultured in 1640 medium supplemented with 10% fetal bovine serum and 1% penicillin-streptomycin at 37 °C in a humidified incubator with 5% CO_2_. After cell attachment, the cell medium was then replaced with 150 μL fresh medium containing Au_8_NCs, R837/BMS NPs, and R837/BMS@Au8 NPs(10 µM). After 24 h of administration, the cells were treated with different doses of X-ray (0.25, 0.5, 1, and 2Gy). After 24 h, CCK-8 reagent (10 μL per well) was added to the wells and incubated for 1 h according to the manufacturer’s protocol. The absorbance at 450 nm was measured using a multimode plate reader. The relative cell viability (%) was calculated as follows:

(A_test_/A_control_) × 100%.

**Determination of Au contents in cancer cells**

4T1 cells were seeded into 96-well plates at a density of approximately 2 × 10^4^ cells per well and cultured in 1640 medium supplemented with 10% fetal bovine serum and 1% penicillin-streptomycin at 37 °C in a humidified incubator with 5% CO_2_. After cell attachment, the cell medium was then replaced with 150 μL fresh medium containing Au_8_NCs, and R837/BMS@Au8 NPs (10 µM) (10 well/group). After 24 h of incubation, two sets of cells were collected and centrifuged. Obtained cell precipitations were dispersed in 5 mL of HNO_3_ solution overnight and then digested with 5 mL of H_2_O_2_ (30%) under boiling conditions at 200 ℃. Use ICP-MS method to determine the content of Au in the samples.

**Colony formation assay**

4T1 cells were seeded in 35 mm plate and incubated overnight for cell attachment. Then, the cells were divided into 2 groups (R837/BMS@Au8 NP and R837/BMS@Au8 NPs + RT). The concentration of R837/BMS@Au8 NPs was 10 µM. After 24 h of administration, the cells were treated with X-ray irradiation at a dose of 1 Gy, and macroscopic cell colonies formed after 14 days. The cell colonies were fixed with 4% paraformaldehyde for 15 min and stained with 0.2% crystal violet for 10 min.

**Live/dead assay**

4T1 cells were cultured on glass-bottomed Petri dishes with an initial density of 10^4^ cells/dish and incubated overnight for cell attachment. Then, the cells were divided into 6 groups (PBS, RT, Au8 + RT, R837/BMS NPs, R837/BMS@Au8 NPs, and R837/BMS@Au8 NPs + RT). After 24 h of administration, the cells were irradiated with X-ray (1 Gy). After another 24 h, the cells were stained using Calcein-AM/PI working solution. Confocal images were taken with a Leica TCS SP8 CLSM (excitated at 488 and 552 nm).

**Intracellular DNA breakage**

4T1 cells were cultured on glass-bottomed Petri dishes with an initial density of 10^3^ cells/dish and incubated overnight for cell attachment. Then, the cells were divided into 4 groups (Au_8_NCs, RT, Au_8_NCs + RT, and R837/BMS@Au8 NPs + RT). After 24 h of administration, the cell medium was removed from the wells and irradiated with X-ray (1 Gy). Another 24 h later, the cells were fixed with 4% paraformaldehyde for 15 min and permeated with 0.25% Triton X-100 for three times. Then, the samples were blocked with 1% bovine serum albumin (BSA) for 1 h and incubated with γ-H2AX antibody overnight at 4℃. Ultimately, the secondar antibody Alexa Fluor™ 647 goat anti-rabbit IgG was added and incubated for 2 h at room temperature, and DAPI was used to stain cell nuclei. The cells were imaged using CLSM (excitated at 405 and 647 nm).

**Intracellular ROS detection**

First, 4T1 cells were seeded in glass-bottomed Petri dishes with an initial density of 10^4^ cells/dish and incubated overnight for cell attachment. Then, the cells were divided into 4 groups (Au_8_NCs, RT, Au_8_NCs + RT, and R837/BMS@Au8 NPs + RT). The culture medium in each dish was removed and then replaced with 1mLculture medium containing Au_8_NC or R837/BMS@Au8 NP (10 µM). After incubation for 24 h, 20 *μ*L of DCFH-DA (0.03 mg/mL) was added to the medium and cultured for another 30 min, followed by irradiation with X-ray (1 Gy). Finally, the cells were washed with PBS and imaged using a fluorescence microscope.

**Isolation of immature DCs**

Murine bone-marrow-derived DCs (BMDCs) were isolated from Balb/c mice. Briefly, bone marrow was collected from the femurs and tibias of the mice according to the established protocols [1,2]. Red blood cells were lysed with an ammonium chloride solution. The remaining cells were washed twice and resuspended in the complete RPMI 1640 medium containing recombinant murine GM-CSF (10 ng/mL). After 48 h of incubation, non-adherent cells were removed, and adherent cells were collected, followed by further incubation for 6 days. Then, non-adherent and loosely adherent cells were harvested and used as immature DCs.

**In vivo antitumor biosafety**

On day 14 after treatments, all mice were sacrificed, and their organs were collected for pathological investigation. The hearts, livers, spleens, lungs, kidneys, and tumors were fixed in 4% paraformaldehyde, embedded in paraffin, sectioned into ~5 μm, and stained with hematoxylin and eosin (H&E).

**References**

1. You J, Zhang Hu F, Du Y, Yuan H, Zhu J, Wang Z, Zhou J, Li C. Pharm.Res*.* 2014;**31**:554−565.

2. You J, Shao R, Wei X, Gupta S, Li C. Small. 2010;6:1022−1031.


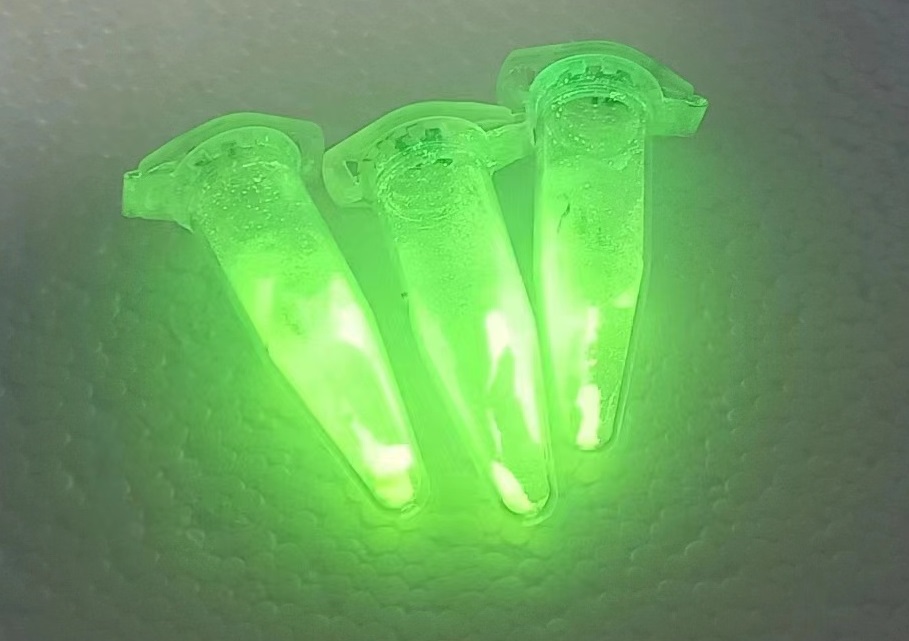


Fig. S1 Photo of Au_8_NC powder under 254 nm ultraviolet lamp irradiation.


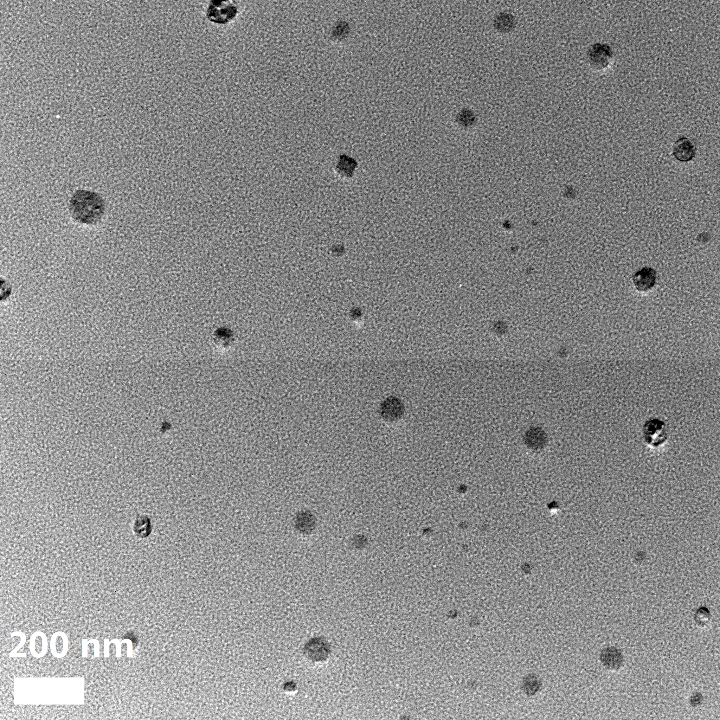


Fig. S2 TEM image of BMS-1 NPs.


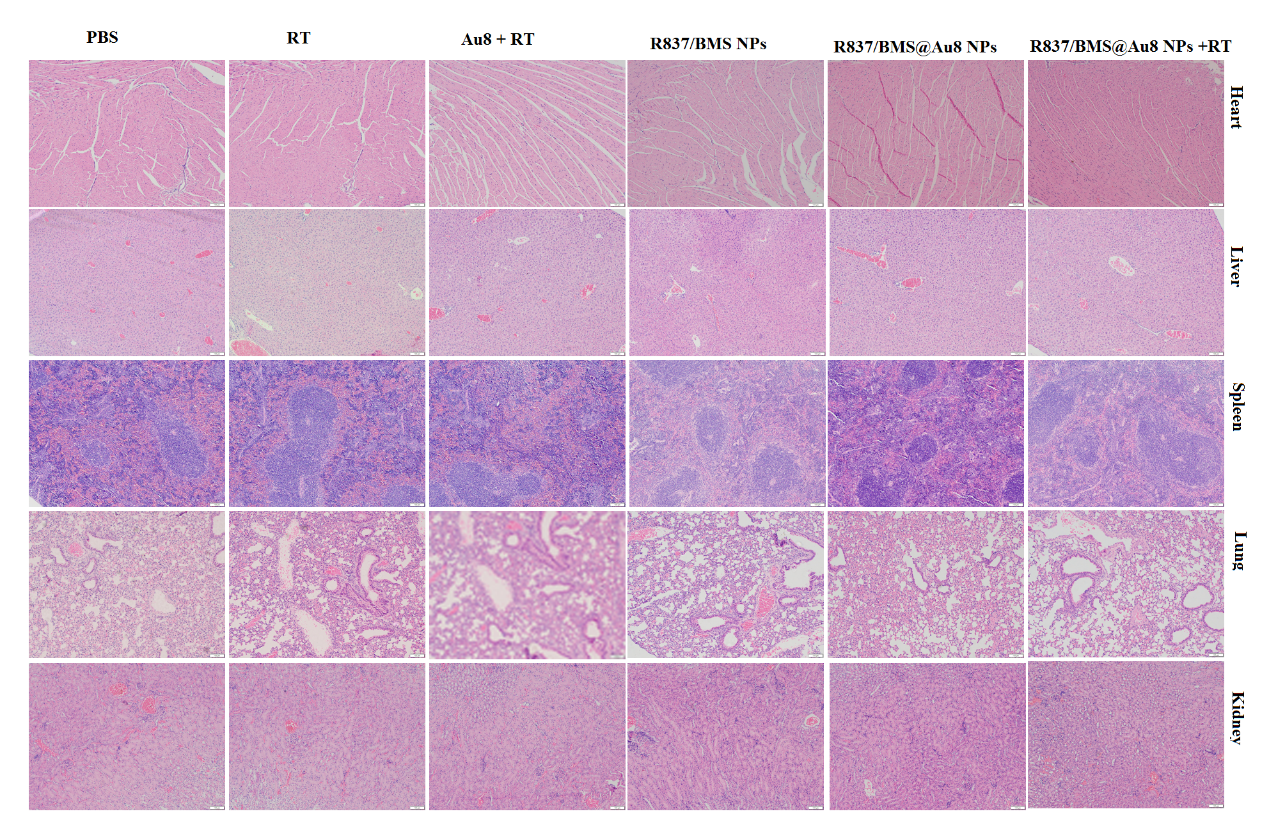


Figure S3. H&E images of heart, liver, spleen, lung and kidney after different treatments.
